# Tuberculosis Presenting As Spontaneous Pneumothorax: A Case Report

**DOI:** 10.7759/cureus.77927

**Published:** 2025-01-24

**Authors:** Jay Bhanushali, Babaji Ghewade, Ulhas Jadhav, Pankaj Wagh, Souvik Sarkar, Arman Sindhu, Bingu Shiv Kiran Reddy, Amit N Toshniwal

**Affiliations:** 1 Respiratory Medicine, Jawaharlal Nehru Medical College, Datta Meghe Institute of Higher Education and Research, Wardha, IND

**Keywords:** air leak syndrome, conservative medical management, intercostal drain, pleural tuberculosis, tension pneumothorax

## Abstract

Secondary spontaneous pneumothorax (SSP) is one of the rare and crucial complications of pulmonary tuberculosis (TB) and can become a fatal condition if it progresses to tension pneumothorax. This case report describes a 32-year-old male who presented with sudden onset chest pain and shortness of breath for the past three hours. On examination, he exhibited tachypnea and hypoxia at rest, and chest imaging revealed a right-sided pneumothorax. Immediate intercostal chest tube insertion was performed and subsequent computed tomography (CT) of the chest was conducted after lung expansion to determine the underlying cause, most likely TB. The patient was subsequently managed conservatively with intercostal chest drain and anti-TB therapy.

## Introduction

A spontaneous pneumothorax generally occurs without prior chest trauma. It can be classified into two types: primary, which occurs in individuals without underlying lung disease, and secondary, which occurs in those with preexisting lung conditions. Secondary spontaneous pneumothorax (SSP) can result from various lung diseases, including pulmonary tuberculosis (TB). In the context of pulmonary TB, a spontaneous pneumothorax may arise due to residual fibrosis, retractions, and bullae formation. The incidence of spontaneous pneumothorax in individuals with active pulmonary TB is estimated to be around 1-2% [1]. Accurate initial assessment and prompt intervention are crucial in preventing hemodynamic deterioration in cases of tension pneumothorax. Treatment typically involves immediate invasive procedures, such as the insertion of a large-bore chest tube and video-assisted thoracoscopic surgery [2]. This case highlights the importance of prompt management in pneumothorax to avoid further hemodynamic compromise due to impending tension pneumothorax. 

## Case presentation

**Figure 1:**
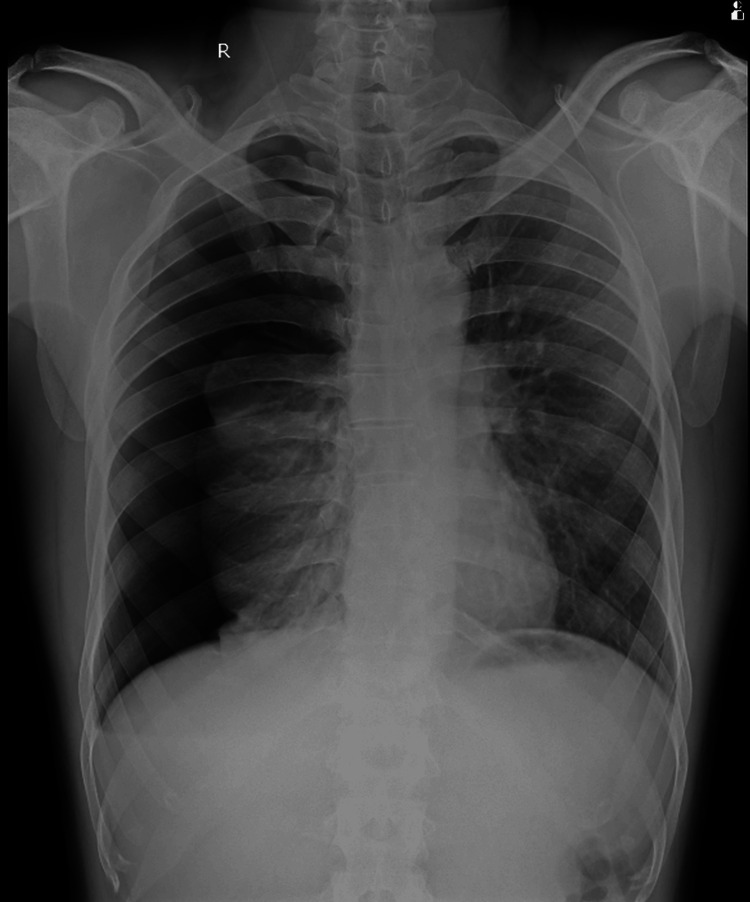
Chest X-ray (PA view) indicating a right-sided pneumothorax. PA, posteroanterior

A 32-year-old tall and thin male presented to the emergency department with a sudden onset of right-sided chest pain and breathlessness for approximately three hours. The patient had no history of trauma and had been otherwise healthy. On examination, he appeared dyspneic and restless but was conscious, oriented, and responsive to commands. His vital signs showed a pulse rate of 118 beats per minute, a respiratory rate of 28 breaths per minute, and a blood pressure of 120/80 mm Hg in both upper limbs. His oxygen saturation was 89% on ambient air, and on percussion, a hyperresonant note was heard over all the areas on the right side of the chest. Auscultation of the right thorax revealed absent breath sounds. Suspecting a pneumothorax, the patient was promptly transferred to radiology for a chest X-ray, which confirmed a right-sided pneumothorax with the underlying lung collapsed toward the hilum (Figure 1).

**Figure 2:**
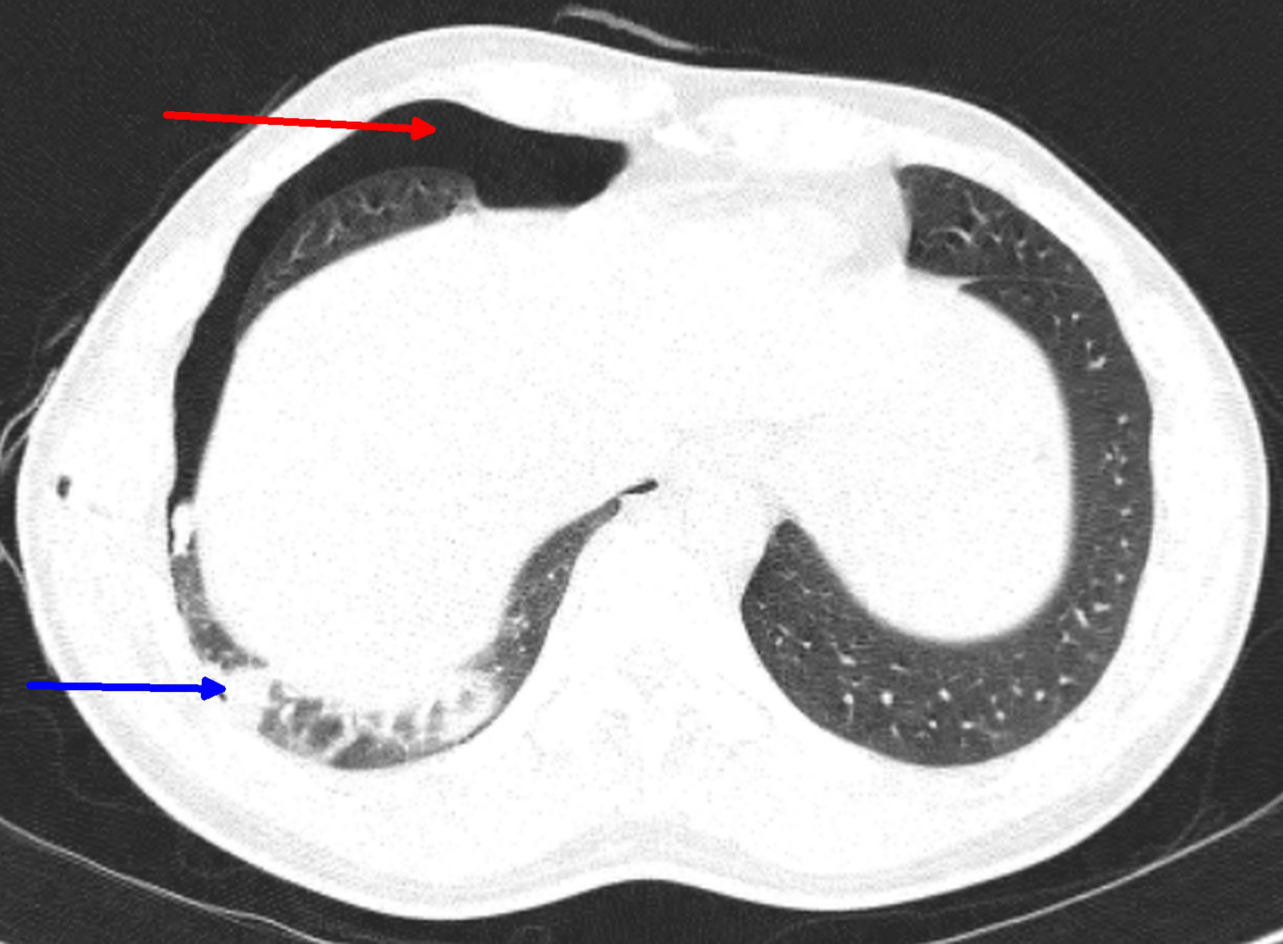
HRCT of chest suggestive of residual pneumothorax (red arrow) and patchy consolidation in the right lower lobe (blue arrow). HRCT, high-resolution computed tomography

Immediate management involved the insertion of an intercostal chest tube, allowing air to be drained. This intervention resulted in a rapid improvement in the patient's condition, with oxygen saturation rising to 96% on room air and resolution of chest pain and breathlessness. A high-resolution computed tomography (HRCT) of the thorax was subsequently performed, revealing residual pneumothorax, a partially collapsed segment of the right lower lobe, and a few subcentimetric lymph nodes in the paratracheal region and aortopulmonary window (Figure 2).

The patient's other lab parameters were suggestive of iron deficiency anemia and raised erythrocyte sedimentation rate (ESR) levels (Table 1).

**Table 1:** Lab parameters upon initial evaluation.

Lab parameters	Observed value	Reference range
Hemoglobin	8.4 g%	13.5-17.5 g%
Mean corpuscular value	64 fL	80-100 fL
Mean corpuscular hemoglobin concentration	290 gm/L	320-360 gm/L
Serum ferritin	38 ng/mL	30-400 ng/mL
Total iron binding capacity	500 mcg/dL	240-450 mcg/dL
Transferrin saturation	11%	15-50%
Erythrocyte sedimentation rate	56 mm/hr	<15 mm/hr

**Figure 3:**
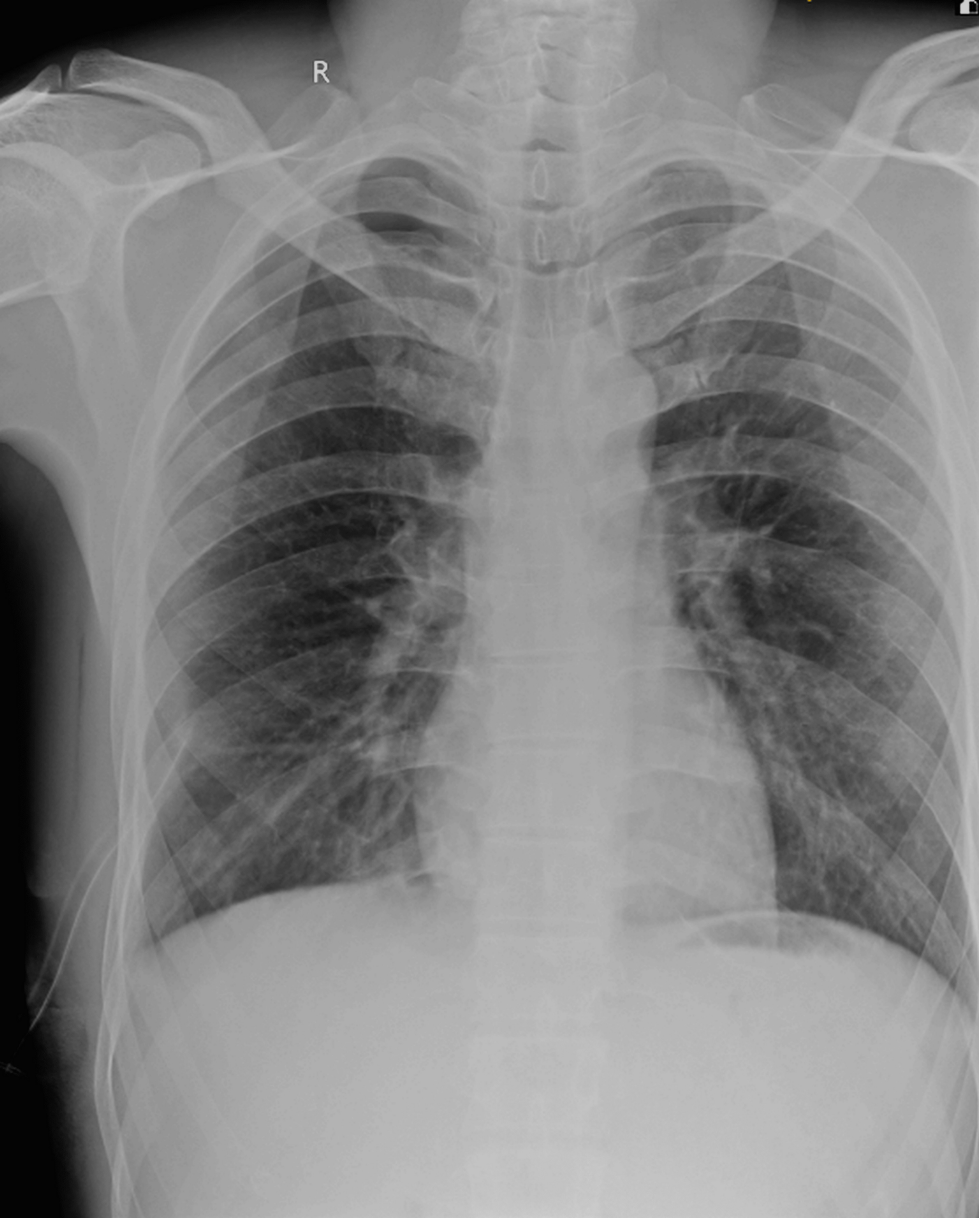
Chest X-ray (PA view) showing complete radiological resolution of pneumothorax with an intercostal drain in situ on the right side. PA, posteroanterior

Patient sputum was sent for cartridge-based nucleic acid testing (CBNAAT) for Mycobacterium tuberculosis, which was negative. Within 48 hours of intercostal chest tube insertion, the patient achieved complete radiological resolution of the pneumothorax and was asymptomatic (Figure 3).

A Mantoux test was also performed, which was positive, suggesting Mycobacterium tuberculosis infection. Considering the positive findings from the history, laboratory results, and imaging, the patient was diagnosed with SSP, likely due to TB.

The intercostal chest tube was removed on the third day, and the patient experienced no immediate recurrence of pneumothorax. He was subsequently discharged on empirical anti-tubercular treatment comprising Rifampicin, Isoniazid, Ethambutol, and Pyrazinamide according to the weight-based regimen.

## Discussion

SSP in the context of TB arises due to the rupture of subpleural bullae or cavities formed as a result of the disease. The chronic inflammatory process in TB leads to fibrosis, retraction, and the formation of bullae, which can rupture and result in pneumothorax [2]. The pathophysiology of SSP involves air leaking into the pleural space, leading to lung collapse and compromised respiratory function. In severe cases, this can progress to tension pneumothorax, causing significant hemodynamic instability.

The patient's presentation with sudden onset chest pain and shortness of breath is typical of pneumothorax. Physical examination findings, including tachypnea, hypoxia, and diminished breath sounds on the affected side, are characteristic of this condition. The prompt use of chest X-ray confirmed the diagnosis of right-sided pneumothorax, which is essential for timely management [3].

Immediate insertion of an intercostal chest tube is the cornerstone of pneumothorax management, particularly in cases at risk of progressing to tension pneumothorax. In this case, the rapid improvement in oxygen saturation and resolution of symptoms following chest tube insertion highlights the efficacy of this intervention [4]. Subsequent HRCT provided detailed insights into the underlying condition, revealing residual pneumothorax and collapsed lung segments but no bullae or blebs, which helped confirm the diagnosis of SSP secondary to TB.

Empirical anti-tubercular therapy was initiated based on clinical presentation and other supportive findings on labs and imaging. The regimen, including Rifampicin, Isoniazid, Ethambutol, and Pyrazinamide, follows standard treatment protocols for TB and aims to address the underlying infection [5]. This approach not only treats TB but also reduces the risk of recurrence of SSP by resolving the primary disease process.

The patient's rapid recovery, with complete radiological resolution of pneumothorax within 48 hours and no recurrence following chest tube removal, is encouraging. It underscores the importance of early intervention and appropriate anti-tubercular therapy in managing SSP. Continuous monitoring and follow-up are crucial to ensure complete recovery and promptly address any complications [6].

Similar cases in the literature emphasize the importance of prompt diagnosis and intervention in SSP. For instance, Briones-Claudett et al. (2020) reported a case where SSP was the initial presentation of TB, underscoring the diverse clinical manifestations of TB and the need for high clinical suspicion [1]. Pradana (2020) also highlighted the critical management steps in TB-associated tension pneumothorax, validating the approaches taken in this case [2].

The occurrence of SSP in the progression of active TB is a recognized phenomenon, though it remains under-researched. Prior studies have indicated a likelihood of 0.6% [7] to 1.4% [8], closely aligning with the 0.95% identified in the current study. This case study suggests that approximately 1% of patients with active TB may experience this issue [9], a consideration that should be integrated into all national TB control programs [10].

## Conclusions

Spontaneous tension pneumothorax associated with TB is a critical condition that demands early detection and swift treatment to reduce mortality. When investigating the cause of spontaneous pneumothorax, it is important not to overlook secondary pneumothorax caused by TB, which requires prompt diagnosis and management.

This case report reinforces the importance of rapid diagnosis and intervention in SSP, mainly in TB patients. It illustrates the clinical pathway from presentation to resolution, emphasizing the critical role of imaging and timely therapeutic interventions. Continuous monitoring and appropriate anti-tubercular therapy are essential components of comprehensive care in such cases, ensuring favorable outcomes and preventing recurrence.
